# pH levels drive bacterial community structure in sediments of the Qiantang River as determined by 454 pyrosequencing

**DOI:** 10.3389/fmicb.2015.00285

**Published:** 2015-04-20

**Authors:** Shuai Liu, Hongxing Ren, Lidong Shen, Liping Lou, Guangming Tian, Ping Zheng, Baolan Hu

**Affiliations:** Department of Environmental Engineering, Zhejiang UniversityHangzhou, China

**Keywords:** pH levels, nitrate concentration, 454 pyrosequencing, bacterial diversity, Qiantang River

## Abstract

The Qiantang River is a typical freshwater ecosystem that acts as an irreplaceable water source in Zhejiang Province in southeastern China. However, the effects of environmental factors on the bacterial community of this freshwater ecosystem have not been determined. In this study, seven sediment samples were collected along the river. Their bacterial communities were identified using 454 high-throughput sequencing, and the primary environmental factors responsible for shaping the community structure were analyzed. The number of bacterial operational taxonomic units (OTUs) ranged from 2637 to 3933. Using a linear-regression analysis, the OTU numbers were significantly positively correlated with pH (*r* = 0.832, *p* < 0.05) and negatively correlated with nitrate concentration (*r* = −0.805, *p* < 0.05). A redundancy analysis (RDA) was also performed to test the relationship between the environmental factors and bacterial community composition. The results indicated that pH (*p* < 0.05) and nitrate concentration (*p* < 0.05) were the most significant factors that determined the community distribution of sediment bacteria.

## Introduction

For decades, microbial ecologists have faced the challenge of inferring the composition of microbial communities from modestly sized ribosomal RNA (rRNA) data sets representing amplicon libraries from environmental DNA (Bowen et al., 2012). Since 2006, 454 pyrosequencing when combined with the well-established b-diversity analytical tools has become a robust tool for analyzing the composition of bacterial communities and their response to environmental changes in a variety of niches (Sogin et al., 2006). It has been applied to analyse the microbial communities in marine water (Qian et al., 2011), lakes (Vila-Costa et al., 2013), soil (Lauber et al., 2009), sewage treatment plants (Zhang et al., 2012), hot springs (Huang et al., 2013), sponges (Giles et al., 2013), bioreactors (Zhu et al., 2012), human hand surfaces (Fierer et al., 2008), and human distal intestines (Claesson et al., 2009). Also, a serious studies using this method have been conducted to investigate the bacterial community structures in lake/reiver sediments. Yergeau et al. (2012) reported the potential impacts of oil sand mining on the neighboring aquatic microbial community structure in the Athabasca River sediment. Bai et al. (2012) explored the bacterial communities in Dianchi (an eutrophic lake) in China and found increasing allochthonous organic carbon could enhance bacterial diversity and biomass in the lake sediment. Liu et al. (2014) presented a detailed outline of the biogeographic patterns of benthic microbial communities in the Pearl Estuary sediments.

Environmental factors can affect the abundances and taxonomic compositions of microbial communities (Allison and Martiny, 2008). Wang et al. (2012) found that the bacterial community variance correlated most strongly with water temperature, conductivity, pH, and dissolved oxygen (DO) content in freshwater, intertidal wetland, and marine sediments. The metal content was found to be the most influential factor shaping the bacterial community composition, structure and diversity in coastal sediment (Sun et al., 2013). Soil pH was responsible for the spatial distribution of bacterial communities with elevation on Changbai Mountain (Shen et al., 2013a), and pH was also found to be the dominating factor driving the variations in archaeal diversity and community structure in tropical soil (Tripathi et al., 2013). Xiong et al. (2012) found that pH was the best predictor of bacterial community structure in alkaline sediments and confirmed that both geographic distance and chemical factors govern bacterial biogeography in lake sediments.

The Qiantang River is a major river system in Zhejiang Province in southeastern China. The total length of the river is 688 km, and the area of its watershed is 55,600 km^2^. The Qiantang River is the main source of industrial, agricultural, and domestic water supplies for Zhejiang Province (Liu et al., 2013b). We have primarily focused on the N-cycle microorganisms, community structures of ammonia-oxidizing archaea (AOA), ammonia-oxidizing bacteria (AOB) (Liu et al., 2013b), anammox bacteria (Hu et al., 2012b), and N-damo bacteria (Shen et al., 2014b) in the freshwater sediment of the Qiantang River as well as the main environmental factors influencing the numbers and distribution of these bacteria. However, we have not examined the entire bacterial community composition in the Qiantang River or determined the main environmental factors shaping the community structure. Thus, the goal of this study was to investigate the diversity and community structure of bacteria in the Qiantang River and identify the principal environmental factors driving the spatial distribution of the bacterial communities along the Qiantang River.

## Materials and methods

### Study sites and sample collection

The Qiantang River is located in southeastern China (28.17° N to 30.48° N and 117.62° E to 121.87° E), and it is an irreplaceable water source to Zhejiang Province. Seven sediment samples (JJY, XY, MC, BQ, ZX, YS, JX) from the upstream (Lanxi) to downstream (Hangzhou) sections of the Qiantang River were collected, which was introduced in our previous work (Liu et al., 2013b). The top 3 cm of the seven sediments samples were carefully obtained using box cores. Each sediment sample was split into two equal parts, with one stored at −80°C for DNA extraction and the other stored at −4°C for further chemical analysis. The methods used determine the physical and chemical properties have been previously described (Shen et al., 2013b). The pH were determined in situ (immediately after the sediments were sampled) using an IQ150 pH meter (IQ Scientific Instruments Inc., Carlsbad, CA, USA). Soil ammonium, nitrite and nitrate were extracted using 2 M KCl. The soil organic carbon content was determined using the K_2_Cr_2_O_7_ oxidation method. Total nitrogen content was determined using the FOSS Appl Microbiol Biotechnol Kjeltec™2300 analyser (FOSS Group, Höganäs, Sweden).

### DNA extraction, PCR amplification and high throughput pyrosequencing

DNA extraction was performed as previously described (Hu et al., 2012a). The extracted genomic DNA was examined in 1.0% agarose gels by electrophoresis and quantified using a NanoDrop ND-1000 spectrophotometer as previously introduced (Hu et al., 2014a). The DNA was stored at −20°C for further PCR amplification, and the primer pairs 357F and 926R were used to amplify the V3-V5 hypervariable regions of bacterial 16S rRNAs (*Escherichia coli* positions 357–926) (Liu et al., 2013a,b). A barcode was permuted for each sample to allow for the identification of individual samples in a mixture within a single pyrosequencing run (Hu et al., 2014b). Each sample was amplified in triplicate with a 20 μ L reaction system using the following protocol: 95°C for 2 min, 25 cycles at 95°C for 30 s, 55°C for 30 s, and 72°C for 30 s, and a final extension at 72°C for 10 min. The three replicate PCR products of each sample were mixed together and purified with an AxyPrep DNA purification kit (AXYGEN). All of the samples were quantified by TBS-380 and mixed at an equimolar ratio in a single tube to be run on a Roche FLX 454 pyrosequencing machine (Roche Diagnostics Corporation, Branford, CT, USA), which produces reads from the forward direction primer 357F.

### Statistical analysis

A bioinformatic analysis was performed using the Mothur software package (http://www.mothur.org) under the standard procedure (Schloss et al., 2009). The sequences obtained were initially screened for their barcodes and primers and only sequences with exact matches were included. The maximum mismatch for both barcodes and primers was zero. Then the sequences with the length less than 200 bp were excluded. Chimeras were detected by using the order of chimera.uchime of Mothur package, and sequences with chimeras were removed (Hu et al., 2014b). After denoising and chimera inspection, the high-quality reads were used to generate a distance matrix and calculate the operational taxonomic units OTUs clustering with a 3% nucleotide cutoff. The high-quality reads were then aligned against the bacterial SILVA database (16S, SSU111), and each sequence was taxonomically classified. By using the command classify OTU in Mothur, each OTU was assigned. Additionally, the diversity index (Chao, Shannon and Simpson index) of the seven samples was estimated.

A composition analysis was conducted on the phylum and class levels, and the sequences assigned to no rank were removed first. The library size of each sample was normalized prior to the composition analysis. The top 20 phyla or classes were identified and analyzed, and a cluster analysis (CA) was performed to reveal the similarity of different samples using the software PAST, which is based on the algorithm of Bray–Curtis at the phylum and class levels. The ecological distributions of the bacterial communities and their correlations with environmental factors were determined using CANOCO software (ter Braak and Šmilauer, 2005). The abundance of each OTU containing more than 10 sequences was used to conduct a principal components analysis (PCA) and a redundancy analysis (RDA). In addition, a Pearson correlation analysis (significance level α = 0.05) was used to test for correlations between the taxonomic diversity and environmental factors (Shen et al., 2014a).

### Accession numbers

The sequences were deposited in GenBank under accession number SRR1118214.

## Results

### Diversity of bacterial communities

After all of the raw sequences had been subjected to quality control processing, including trimming and filtering, the low quality sequences were removed to yield a total of 58892 high-quality sequences for the seven sediment samples. The average library size was 8413 sequences, and the OTU numbers and diversity indices of the seven samples were calculated at the 3% cutoff level and are summarized in Table S1. Plots of the OTU numbers versus sequence numbers, also known as the rarefaction curves, are shown in Supplementary Figure S1. The OTU numbers of the seven sediments ranged from 2637 to 3933, with the sediment from ZX having the richest diversity (3933 OTUs), followed by the sediment samples from JX (3627 OTUs) and JJY (3614 OTUs). The sediment from XY only had 2637 OTUs and showed the lowest diversity. The results of the Ace, Chao and Shannon indices were similar regarding the OTU number.

### Bacterial community composition

By normalizing the library size to 6748 sequences, the bacterial community compositions of the seven sediment samples were analyzed at two different taxa levels (phylum and class levels), although a proportion of the high-quality sequences could not be assigned to any taxa at the two levels (from 11.0 to 14.7% at the phylum level and from 16.1 to 21.8% at the class level).

**Figure 1:**
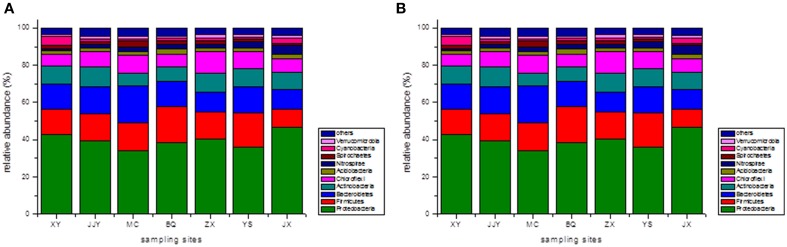
**The relative abundances of different phyla (A) and classes (B) in the seven sediment samples along the Qiantang River**.

At the phylum level, the top 10 phyla were selected, and the remaining sequences were assigned to a cluster named “the others” (Figure 1). The results revealed that Proteobacteria was the most abundant phylum across all seven sediment samples. The proportions of Proteobacteria in samples ranged from 33.99% at site MC to 46.70% at site JX. The other dominant phyla were Firmicutes (9.71–19.07%, averaging 14.97%), Bacteroidetes (10.59–20.08%, averaging 13.94%), Actinobacteria (6.82–10.85%, averaging 9.15%), and Chloroflexi (6.34–11.75%, averaging 8.33%). These five phyla dominated (83.50–87.45%) the bacterial communities in all seven sediment samples, and the following phyla were present at less than 3%: Nitrospirae (averaging 2.44%), Acidobacteria (averaging 2.23%), Cyanobacteria (averaging 2.11%), Spirochaetes (averaging 1.88%), and Verrucomicrobia (averaging 1.35%). The abundance of Nitrospirae-related sequences detected at site JX (4.78%) was much higher than at the other sites, and the Cyanobacteria-related sequences found at site XY (5.20%) were at least two times higher than at the other sites.

In addition to at the phylum level, the bacterial community compositions were also analyzed at the class level. Similarly, the top 10 classes of bacteria were selected, and the relative abundances of the different classes of bacteria are shown in Figure 1. The five dominant classes were *Clostridia* (7.53–14.10, averaging 11.70%), *Beta-proteobacteria* (10.17–13.58%, averaging 11.69%), *Delta-proteobacteria* (8.87–14.50%, averaging 11.25%), *Gamma-proteobacteria* (6.76–12.71%, averaging 10.37%), and *Alpha-proteobacteria* (5.29–11.76%, averaging 8.58%). Within the Proteobacteria group, *Beta-proteobacteria* was the most dominant class, followed by *Delta-proteobacteria*, *Gamma-proteobacteria*, and *Alpha-proteobacteria*. The *Gamma-proteobacteria*-related sequences at the sites JJY (8.66%) and MC (6.76%) were much lower than in the other sites, and the proportion of *Alpha-proteobacteria*-related sequences at the site JJY (11.76%) were significantly higher than at the other sites.

### Similarity analysis of the seven sediment samples

**Figure 2:**
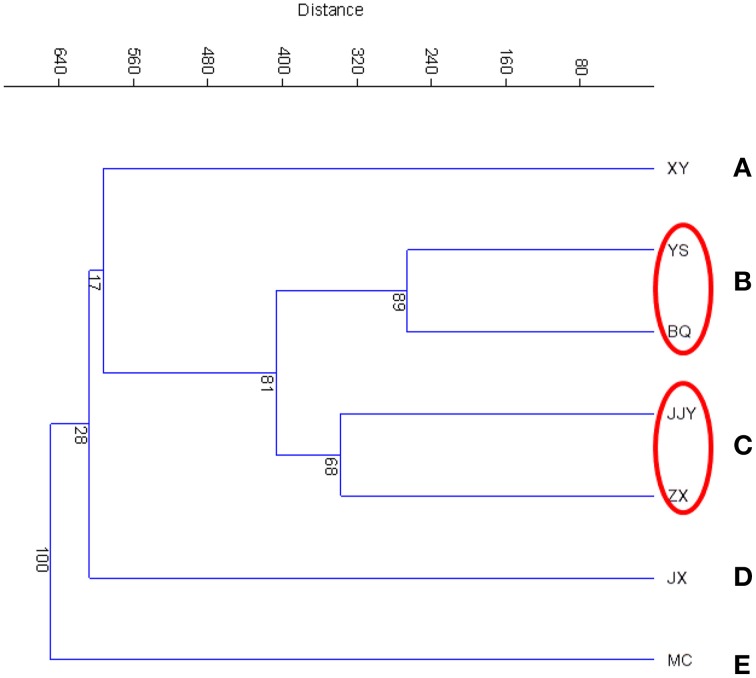
**Cluster analysis at the phylum level based on the Bray–Curtis distances of the seven sediment samples along the Qiantang River**.

The similarity of the seven sediment samples was evaluated according to a cluster analysis (CA) and principal components analysis (PCA). The cluster analysis of the seven sediment samples was conducted at the phylum and class levels, which are shown in Figure 2 (phylum level) and Figure S2 (class level). The results showed that at the phylum level, the seven samples were clustered into five groups. Group B and group C each contained two sites, with sites YS and BQ included in group B and JJY and ZX included in group C. The other three groups contained only one sampling site each. A similar grouping pattern was also found at the class level (Figure S2). A PCA was also conducted to analyze the similarity of the seven sediment samples (Figure S3). Similar to the result of CA, in the PCA analysis (Figure S3), YS and BQ were grouped into the same cluster, JJY and ZX were grouped into the same cluster.

### Relationship between the environmental factors and bacterial community diversity

**Figure 3:**
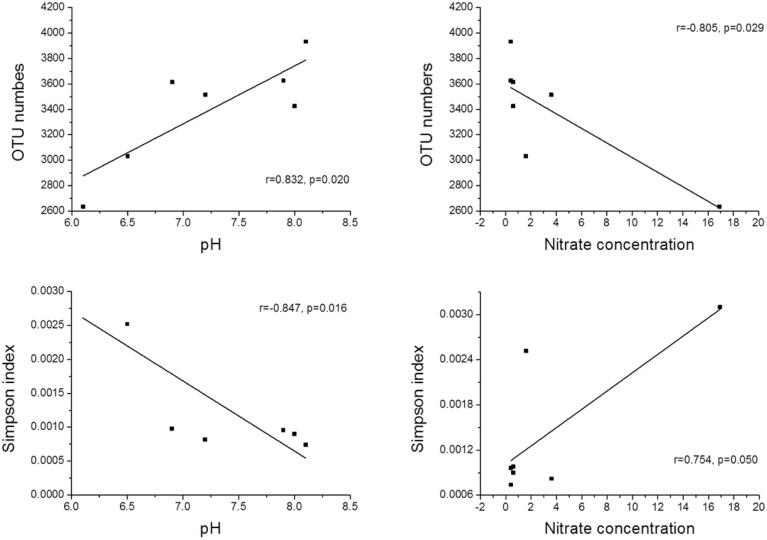
**The relationship between pH, nitrate and bacterial α-diversity**.

**Figure 4:**
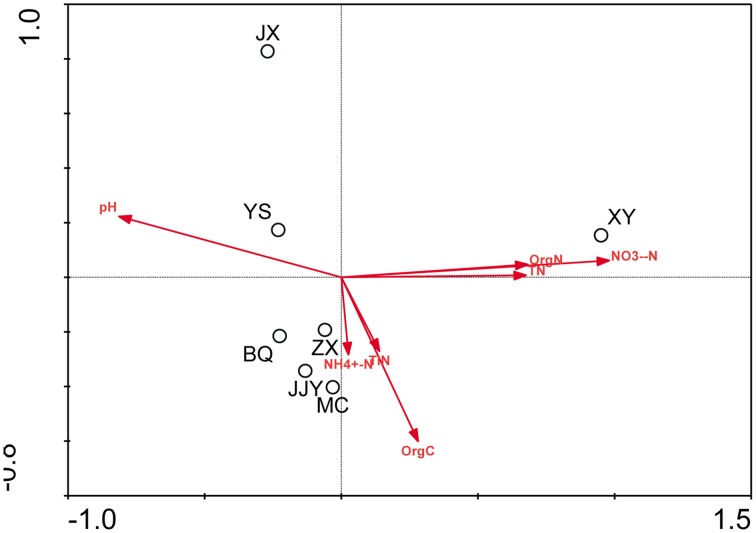
**RDA ordination plots of the relationships between bacterial communities and environmental factors**.

The physicochemical properties of the sediment samples are presented in Table S2. A Pearson analysis (Table S3) and simple linear regression (Figure 3) were used to examine the relationship between the environmental factors and bacterial biodiversity. The pH level was significantly positively correlated with the OTU numbers (*r* = 0.832, *p* < 0.05), whereas the Shannon and Simpson indices were positively (*r* = 0.856, *p* < 0.05) and negatively (*r* = −0.847, *p* < 0.05) correlated with pH, respectively. Another important factor influencing the OTU numbers was the nitrate concentration. The OTU numbers increased as the nitrate concentration decreased (*r* = −0.805, *p* < 0.05). Furthermore, the ammonia concentration (NH^+^_4_-N) (*r* = −0.764, *p* < 0.05) and total inorganic nitrogen (TIN) content (*r* = −0.826, *p* < 0.05) were significantly negatively correlated with the Shannon index. An RDA was also performed to test the relationship between the environmental factors and bacterial community composition (Figure 4), and the results indicated that the pH level was the most significant factor influencing the community distribution of sediment bacteria (*p* < 0.05). In addition, the nitrate concentration (NO^−^_3_-N) was also found to significantly influence the bacterial community composition (*p* < 0.05).

## Discussion

The present study provides information on the distribution, diversity and composition of the bacterial communities along the Qiantang River, China. Within the seven examined sediment samples, 2637-3933 OTUs were obtained using a 3% cutoff level, for an average of 3398 OTUs per sample. The bacterial diversity was much lower than that found in a previous study of the bacterial communities of freshwater sediment (Ligi et al., 2013), which reported the discovery of 8331 bacterial OTUs with 97% sequence similarity along the Pearl River. Additionally, the number of OTUs found in this study was lower than that found in soil ecosystems (more than 4000 OTUs) (Roesch et al., 2007). Considering the present sequencing depth (coverage of clone library ranging from 91.8 to 93.5%), the bacterial diversity in the Qiantang River may be underestimated to certain extent because the rarefaction curve did not reach a plateau. Even at the present sequencing depth, the bacterial diversity found in the Qiantang River was higher than what was obtained in hypersaline lake sediments in the USA (Hollister et al., 2010), saline lake sediments in Southern Australia, and alkaline lake sediments in China (Xiong et al., 2012) and the western Arctic Ocean (Kirchman et al., 2010). The environmental pressure in the saline lakes and ocean was much higher than in the freshwater sediment, and this difference is reflected in the increased bacterial diversity in the conditions that presented lower environmental pressure.

The bacterial composition along the Qiantang River sediments showed that Proteobacteria was the dominant phylum across all seven sediment samples, with proportions ranging from 33.99% at the site MC to 46.70% at the site JX, with an average proportion of 39.63% across all sites. This result was consistent with previous studies in different ecosystems. Ligi found that Proteobacteria composed 22.7–59.2% of all of the sequences in the freshwater sediments (Ligi et al., 2013), and Roesch et al. (2007) reported that more than 40% of the soil sequences were Proteobacteria. In a study of sewage treatment plants, Proteobacteria was the most abundant phylum in all of the sludge samples (accounting for 36–65% for all of the sequences). Nemergut et al. (2011) examined the global patterns of bacterial communities from various habitats and found that the average level occupied by Proteobacteria in the bacterial population was as high as 40%. In addition to Proteobacteria, the other four most abundant phyla were Firmicutes (9.71–19.07%, averaging 14.97%), Bacteroidetes (10.59–20.08%, averaging 13.94%), Actinobacteria (6.82–10.85%, averaging 9.15%) and Chloroflexi (6.34–11.75%, averaging 8.33%). The identification of Firmicutes as the second most prevalent phylum was consistent with a study of Brazilian mangrove sediments, which showed that the proportion Firmicutes occupied in the population ranged from 10.5 to 13.8% (Andreote et al., 2012). In metal-contaminated lake sediments, Firmicutes-related sequences could compose nearly 50% of all of the discovered bacteria, and the high concentration of trace mental contamination in the lakes may have been responsible for the high abundance of the endospore-forming Firmicutes (Sauvain et al., 2013). It was notable that in this study, approximately 10% of the sequences were *Clostridia*, which is a class within the phylum Firmicutes. Bacteria in the class *Clostridia* can produce endospores under harsh conditions. Future research should focus on the relationship between the abundance of Firmicutes and possible contamination of the Qiantang River. The fact that Firmicutes prefer eutrophic conditions may be another possible explanation for the appearance of a large number of Firmicutes-related sequences (Zeng et al., 2012). However, in other studies, the proportion of the bacterial communities that Firmicutes occupied was less than 5% (Song et al., 2013; Sun et al., 2013). The relative abundance of Acidobacteria in the Qiantang River was low (1.83–2.38%), which was inconsistent with a series of studies on soils and sediments (Chu et al., 2010; Nemergut et al., 2011). However, numerous studies have suggested that the abundance of Acidobacteria is significantly correlated with pH, and its abundance specifically increases when the pH is lower than 5.5 (Jones et al., 2009). The lowest pH value in the Qiantang River was 6.5. Therefore, it was reasonable that the relative abundance of Acidobacteria was low.

A redundancy analysis showed that the pH level (*p* < 0.05) was the most important environmental factor influencing the distribution and community structure of the bacteria. A linear regression analysis also revealed that the pH level (*p* < 0.05) was significantly positively correlated with bacterial diversity. All of the results showed that pH was the most important factor in determining the diversity and bacterial community differences along the Qiantang River. These findings were consistent with a previous study of freshwater sediment (Ligi et al., 2013). Ligi et al. (2013) reported that variations in the composition of the bacterial community within wetland sediments were related to pH, with the pH of wetlands ranging from 5.44 to 7.16. In addition to freshwater sediments, the pH level dominated the diversity and community of bacteria in other ecosystems, including soils (Shen et al., 2013a), hypersaline sediments (Hollister et al., 2010) and glacier-fed streams (Wilhelm et al., 2013). Moreover, the pH level is an important determinant of bacterial community structure in acidic, neutral and alkaline soils. A recent comprehensive analysis of the biogeography of soil bacterial communities in the Arctic showed that the soil bacterial community composition and diversity were structured according to local variations in the soil pH (4.0–8.0) rather than geographical proximity to neighboring sites (Chu et al., 2010). In lake sediments with pH values ranging from 6.88 to 10.37, the bacterial community structure, phylotype richness and phylogenetic diversity were primarily correlated with a single parameter: sediment pH (Xiong et al., 2012).

There are at least two general explanations that may explain, either alone or in combination, why soil pH was the best predictor of community composition and diversity across the range of samples included in this study. First, soil pH may not directly alter the bacterial community structure but may instead function as an integrating variable that provides an integrated index of the soil conditions. There are a number of soil characteristics (e.g., nutrient availability, cationic metal solubility, organic C characteristics, soil moisture regimen, and salinity) that are often directly or indirectly related to soil pH, and these factors may drive the observed changes in the community composition because the hydrogen ion concentration varies by many orders of magnitude across the sediments in this study. A second hypothesis is that pH directly imposes a physiological constraint on the soil bacteria by altering competitive outcomes or reducing the net growth of individual taxa that are unable to survive if the soil pH falls outside a certain range (Lauber et al., 2009). Previous studies have reported that pH was linearly correlated with the relative abundance of the main phyla (Chu et al., 2010; Shen et al., 2013a). This phenomenon was not detected in the present study because the range of pH in the sediments from the Qiantang River was small (pH ranging from 6.5 to 8.1). The nitrate concentration was also found to influence the bacterial diversity and community composition and was similar to what was observed in a previous study of freshwater sediments (Ligi et al., 2013). The nitrate concentration may affect the communities of denitrifying and anammox bacteria in the anoxic sediments of the Qiantyang River (Hu et al., 2012b; Shen et al., 2014b).

### Conflict of interest statement

The authors declare that the research was conducted in the absence of any commercial or financial relationships that could be construed as a potential conflict of interest.
