# Supplementary material for: pH levels drive bacterial community structure in sediments of the Qiantang River as determined by 454 pyrosequencing
Source: Front Microbiol. 2015 Apr 20;6:285. doi: 10.3389/fmicb.2015.00285 (PMC4403504; doi:10.3389/fmicb.2015.00285)
Supplement: Supplementary file 1 [file DataSheet1.PDF]

Supplementary information

Table S1. Sequences obtained and the diversity index of the seven samples from the Qiantang River

Sample	No. of seqs	No. of OTUs	Ace	Chao	Shannon	Simpson
BQ	9131	3033	9685	6105.4	7.05	0.00252
JJY	8457	3614	1180	8068.5	7.65	0.00098
JX	9072	3627	1077	7195.4	7.62	0.00096
MC	8223	3514	1156	7804.9	7.60	0.00082
XY	7670	2637	8456	5807.1	7.04	0.00310
YS	7818	3425	1078	7311.7	7.63	0.00090
ZX	8521	3933	1239	8610.1	7.82	0.00074

Table S2. The basic physical and chemical parameters of the sediments collected from the Qiantang River

Sample	pH	OrgC (mg kg^{-1})	TN (mg kg^{-1})	OrgN (mg kg^{-1})	TIN (mg kg^{-1})	NH_4^+-N (mg kg^{-1})	NO_3^--N (mg kg^{-1})
JX	7.9	18.9	623.8	583.5	52.6	52.0	0.4
YS	8.0	13.4	480.3	438.7	41.6	41.0	0.6
ZX	8.1	20.2	173.8	136.5	37.4	37.0	0.4
BQ	6.5	22.4	571.0	387.5	183.6	182.0	1.6
MC	7.2	34.2	561.3	503.7	57.6	54.0	3.6
XY	6.1	26.3	1491.6	1385.6	105.9	89.0	16.9
JJY	6.9	31.5	1274.1	1177.5	96.6	96.0	0.6

Table S3. Pearson correlation analysis of environmental factors and diversity index

r / Environmental factors	OTU number	Shannon index	Simpson index	Chao	Ace
pH	**0.832***	**0.856***	**-0.847***	0.701	0.703
OrgC	-0.098	-0.104	0.072	0.107	0.100
TN	-0.623	-0.501	0.557	-0.466	-0.548
OrgN	-0.569	-0.422	0.488	-0.406	-0.501
TIN	-0.646	**-0.826***	**0.763***	-0.674	-0.580
NH_4^+-N	-0.563	**-0.764***	0.689	-0.611	-0.502
NO_3^--N	**-0.805***	-0.664	**0.754***	-0.642	**-0.757***

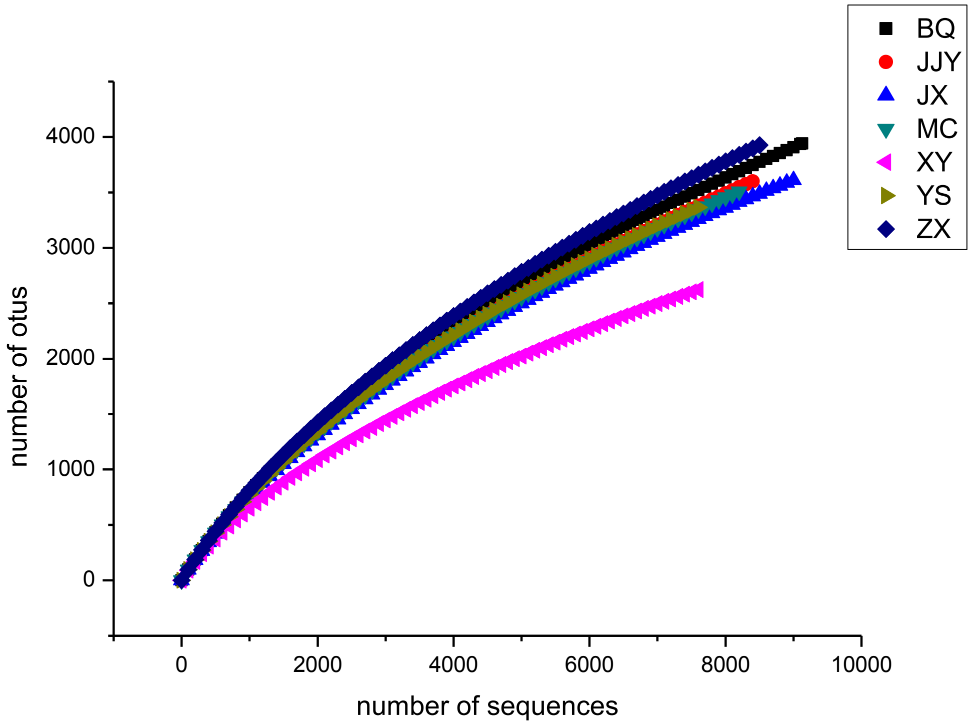

Fig. S1. The rarefaction curve of the seven samples at cutoff levels of 3%

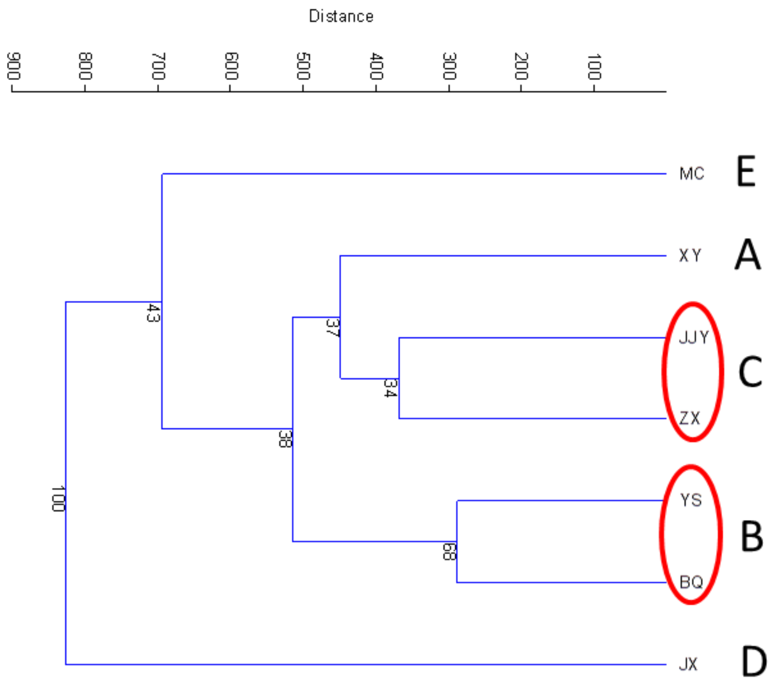

Fig. S2. CA at class level based on Bray–Curtis distances of the seven sediment samples along the Qiantang River

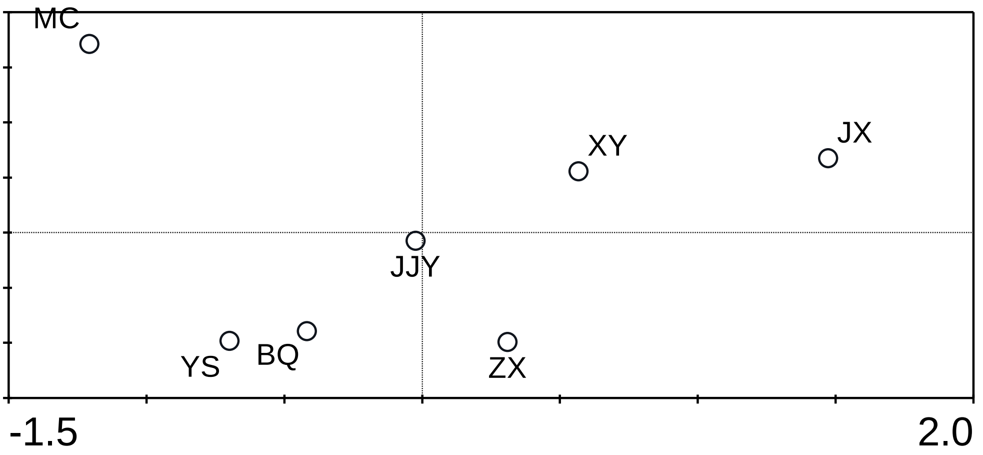

Fig. S3. PCA ordination diagrams of the bacterial community detected in the sediment samples along the Qiantang River
